# Supplementary material for: Examining the Acceptability of Helminth Education Packages “Magic Glasses Lower Mekong” and “Magic Glasses Opisthorchiasis” and Their Impact on Knowledge, Attitudes, and Practices Among Schoolchildren in the Lower Mekong Basin: Protocol for a Cluster Randomized Controlled Trial
Source: JMIR Res Protoc. 2024 Sep 16;13:e55290. doi: 10.2196/55290 (PMC11443236; doi:10.2196/55290)
Supplement: Multimedia Appendix 1 [file resprot_v13i1e55290_app1.docx]

**Multimedia Appendix. Anticipated delivery dates for baseline, intervention delivery, reinforcement and follow-up** **for the “Magic Glasses Lower Mekong” and “Magic Glasses Opisthorchiasis” cluster-randomized controlled trial in Cambodia, Lao PDR, and Thailand.**

|  | Country | | |
| --- | --- | --- | --- |
|  | **Cambodia** | **Lao PDR** | **Thailand** |
| Baseline and intervention delivery | January 2024 | November 2023 | September 2023 |
| Reinforcement (6-8 weeks after intervention delivery) | February/March 2024 | January 2024 | November 2023 |
| Follow-up | September 2024 | August 2024 | July 2024 |
