# Supplementary material for: Examining the Acceptability of Helminth Education Packages “Magic Glasses Lower Mekong” and “Magic Glasses Opisthorchiasis” and Their Impact on Knowledge, Attitudes, and Practices Among Schoolchildren in the Lower Mekong Basin: Protocol for a Cluster Randomized Controlled Trial
Source: JMIR Res Protoc. 2024 Sep 16;13:e55290. doi: 10.2196/55290 (PMC11443236; doi:10.2196/55290)
Supplement: Multimedia Appendix 2 [file resprot_v13i1e55290_app2.docx]

**Multimedia Appendix. OV KAP questionnaire for the “Magic Glasses Lower Mekong” and “Magic Glasses Opisthorchiasis” cluster-randomized controlled trial.**

| **A. SURVEY IDENTIFICATION Date:** (yyyy-mm-dd) \|__\|__ \|__\|__\| / \|__\|__\| / \|__\|__\|  ***To be filled in by research team:*** | | | | | |
| --- | --- | --- | --- | --- | --- |
| **1** | Village (Name and Code) | | | \|__\|__\| | |
| **2** | School (Name and Code) | | | \|__\|__\| | |
| **3** | Grade | | |  | |
| **4** | Class/Section | | | \|__\|__\| | |
| **5** | Student Number | | | \|__\|__\| | |
| **B. PERSONAL INFORMATION** | | | | | |
| 1 | First Name | | |  | |
| 2 | Last Name | | |  | |
| 3 | Sex | | | ☐ 1 – Male ☐ 2 – Female | |
| 4 | Date of Birth (yyyy-mm-dd)  If you can’t remember, use 6 for the month/15 for the day of the month | | | \|__\|__ \|__\|__\| / \|__\|__\| / \|__\|__\| | |
| **C. KNOWLEDGE ABOUT LIVER FLUKE**  *Please let us know what you have heard and what you know about liver fluke.* | | | | | |
| 1 | | Have you ever heard about the liver fluke or Opisthorchiasis? | | | ☐ 0= No (Go to E1)  ☐ 1= Yes  ☐ 2= Don’t know (Go to E1) |
| 2 | | If yes, where did you hear about liver fluke? | | | ☐ 1 = Friend  ☐ 2 = Poster  ☐ 3 = TV  ☐ 4 = Radio  ☐ 5 = Book  ☐ 6 = Brochure  ☐ 7 = School  ☐ 8 = Nurse/Doctor  ☐ 9 = Internet/social media  ☐ 10 = Parents/Family  ☐ 11 = Others, please specify ________________  ☐ 12 = Unknown/Can’t remember |
| 3 | | Have you ever had liver fluke | | | ☐ 0= No  ☐ 1= Yes  ☐ 2= Don’t know |
| 4 | | Do you know somebody who had liver fluke? | | | ☐ 0= No  ☐ 1= Yes  ☐ 2= Don’t know |
| **D. TRANSMISSION, SYMPTOMS AND TREATMENT OF LIVER FLUKES** | | | | | |
| 1 | | How can you get liver fluke? | | | ☐ 1= Mosquito bite  ☐ 2= Swimming in the river/canal  ☐ 3= Fishing  ☐ 4= Playing with soil  ☐ 5= Dirty hands  ☐ 6= Eating raw/undercooked and fermented fish  ☐ 7= Others, specify  ☐ 8= Don’t know |
| 2 | | Do you think liver fluke can make you sick? | | | ☐ 0= No  ☐ 1= Yes  ☐ 2= Don’t know |
| 3 | | What happens if you have liver fluke? | | | ☐ 1= Liver fluke cancer  ☐ 2= Blindness  ☐ 3= Fever  ☐ 4= High blood pressure  ☐ 5= Feeling tired  ☐ 6= Slow growth  ☐ 7= Others, specify:  ☐ 8= Don’t know |
| 4 | | How can you prevent/avoid liver fluke? | | | ☐ 0= Don’t know  ☐ 1= Using latrine  ☐ 2= Sleeping under a mosquito net  ☐ 3= Doing exercise  ☐ 4= Better sewerage system  ☐ 5= Avoid consumption of raw/undercooked fish  ☐ 6= Others, specify: |
| 5 | | Do you think liver fluke can be treated? | | | ☐ 0= No  ☐ 1= Yes  ☐ 2= Don’t know |
| 6 | | If yes, where can you go for treatment | | | ☐ 0= Don’t know  ☐ 1= School  ☐ 2= Health Center  ☐ 3= Hospital  ☐ 4= Traditional or Faith Healer |
| 7 | | What do you think is the treatment for the liver fluke? | | | ☐ 0= Don’t know  ☐ 1= Aspirin  ☐ 2= Praziquantel  ☐ 3= Albendazole  ☐ 4= Others, specify: |
| **E. ATTITUDE ABOUT LIVER FLUKE** | | | | | |
| 1 | | Do you believe that you are likely to be infected with liver fluke? | | | ☐ 0= No  ☐ 1= Yes  ☐ 2= Don’t know |
| 2 | | If yes, what is your risk or chance of getting liver fluke? | | | ☐ 0= None  ☐ 1= Low Possibility  ☐ 2= Medium Possibility  ☐ 3= High Possibility |
| 3 | | Would you be worried if you get infected with liver fluke? | | | ☐ 0= No  ☐ 1= Yes  ☐ 2= Don’t know |
| 4 | | What do you think is the chance that your neighbour will get infected with liver fluke? | | | ☐ 0= None  ☐ 1= Low  ☐ 2= Medium  ☐ 3= High  ☐ 4= Don’t know |
| 5 | | How bad do you think liver fluke is as a disease in your village? | | | ☐ 0= Not severe  ☐ 1= Low severity  ☐ 2= Medium severity  ☐ 3= High severity  ☐ 4= Don’t know |
| 6 | | Liver fluke is a problem in your village | | | 0= Strongly disagree  1=Disagree  2= Agree  3= Strongly Agree |
| 7 | | Have you ever been treated for liver flukes? | | | ☐ 0= No  ☐ 1= Yes  ☐ 2= Don’t know |
| 7.1 | | If yes, how many times? | | | ☐ 1= Once  ☐ 2= Twice  ☐ 3= Three times  ☐ 4= More than 3 times (specify:________)  ☐ 5= Don’t know |
| 7.2 | | If yes, where did you go for treatment? (multiple answers possible) | | | ☐ 1= Health Center  ☐ 2= Hospital  ☐ 3= Traditional or Faith Healer  ☐ 4= Local grocery  ☐ 5= Others, specify: ___________________ |
| 8 | | When was your last treatment with PZQ? | | | 1=Less than 1 month  2=1-6 months  3=7-12 months  4=> 1 year |
| **F. HEALTH EDUCATION RELATED TO LIVER FLUKE** | | | | | |
| 1 | | Has your teacher already told you about liver flukes? | | | ☐ 0= No  ☐ 1= Yes  ☐ 2= Don’t know |
| 2 | | Have you watched video (in social media, Youtube) on liver flukes? | | | ☐ 0= No  ☐ 1= Yes  ☐ 2= Don’t know |
| 3 | | Have you ever done an assignment on liver flukes? | | | ☐ 0= No  ☐ 1= Yes  ☐ 2= Don’t know |
| 4 | | Have you told your parents, sisters and brothers about liver flukes? | | | ☐ 0= No  ☐ 1= Yes  ☐ 2= Don’t know |
| 5 | | Have your parents told you about the liver flukes? | | | ☐ 0= No  ☐ 1= Yes  ☐ 2= Don’t know |
| **G. BEHAVIOUR ABOUT LIVER FLUKE** | | | | | |
| 1 | | | Do you eat raw or live fish? | | ☐ 0= No  ☐ 1= Yes  ☐ 2= Don’t know |
| 2 | | | If yes, how often do you consume raw fish?  (for example, koi pla or other raw fish dishes) | | ☐ 0= Never  ☐ 1= once a year  ☐ 2= 1-3 per month  ☐ 3= 1-3 per week  ☐ 4= 4-6 per week  ☐ 5= Every day or ≥ 1 per day |
| 3 | | | Does your family consume raw undercooked/fermented fish? | | ☐ 0= No  ☐ 1= Yes  ☐ 2= Don’t know |
| 4 | | | How often does your family prepare or consume raw fish? | | ☐ 1= Never or less than once per month  ☐ 2= 1-3 per month  ☐ 3= 1-3 per week  ☐ 4= 4-6 per week  ☐ 5= Every day or ≥ 1 per day |
| 5 | | | How does your family dispose your waste food? | | ☐ 1= Disposal by giving it to dogs or cats  ☐ 2= Discarding to an open pit to which animals had access  ☐ 3= Compost pit at home  ☐ 4= Others, specify? |
