# Supplementary material for: Examining the Acceptability of Helminth Education Packages “Magic Glasses Lower Mekong” and “Magic Glasses Opisthorchiasis” and Their Impact on Knowledge, Attitudes, and Practices Among Schoolchildren in the Lower Mekong Basin: Protocol for a Cluster Randomized Controlled Trial
Source: JMIR Res Protoc. 2024 Sep 16;13:e55290. doi: 10.2196/55290 (PMC11443236; doi:10.2196/55290)
Supplement: Multimedia Appendix 3 [file resprot_v13i1e55290_app3.docx]

**Multimedia Appendix. STH KAP questionnaire for the “Magic Glasses Lower Mekong” and “Magic Glasses Opisthorchiasis” cluster-randomized controlled trial.**

| **A. SURVEY IDENTIFICATION Date:** (yyyy-mm-dd) \|__\|__ \|__\|__\| / \|__\|__\| / \|__\|__\|  ***To be filled in by research team:*** | | | | | | | | | |
| --- | --- | --- | --- | --- | --- | --- | --- | --- | --- |
| 1 | | Village (Name and Code) | \|__\|__\| | | | | | | |
| 2 | | School (Name and Code) | \|__\|__\| | | | | | | |
| 3 | | Grade |  | | | | | | |
| 4 | | Class/Section | \|__\|__\| | | | | | | |
| 5 | | Student Number | \|__\|__\| | | | | | | |
| **B. PERSONAL INFORMATION**  To be filled in by children: Please fill in this questionnaire truthfully with the help of your teacher. All responses will be kept confidential and will not be used against you in any way. The info will be used for research purposes only | | | | | | | | | |
| 1 | | First Name |  | | | | | | |
| 2 | | Last Name |  | | | | | | |
| 3 | | Sex | ☐ 1 – Male ☐ 2 – Female | | | | | | |
| 4 | | Date of Birth (yyyy-mm-dd)  If you can’t remember, use 6 for the month/15 for the day of the month | \|__\|__ \|__\|__\| / \|__\|__\| / \|__\|__\| | | | | | | |
| **C. KNOWLEDGE ABOUT INTESTINAL WORMS** | | | | | | | | | |
| 1 | | Have you ever heard about worms? | ☐ 0 – No ***(Go to F1)*** | | | | | | |
|  |  |  | ☐ 1 – Yes ***(Go 1.a)*** | | | | | | |
|  |  |  | ☐ 2 – Don’t know ***(F1)*** | | | | | | |
|  |  | 1.a Roundworms | ☐ 0 – No ☐ 2 –Yes ☐ 3 - Don’t know | | | | | | |
|  |  | 1.b. Whipworms | ☐ 0 – No ☐ 2 –Yes ☐ 3 - Don’t know | | | | | | |
|  |  | 1.c. Hookworms | ☐ 0 – No ☐ 2 –Yes ☐ 3 - Don’t know | | | | | | |
| 2 | | If you have heard of it, where? (You can choose more than one answer) | ☐ 1 = Friend | | | | | | |
|  |  |  | ☐ 2 = Poster | | | | | | |
|  |  |  | ☐ 3 = TV | | | | | | |
|  |  |  | ☐ 4 = Radio | | | | | | |
|  |  |  | ☐ 5 = Book | | | | | | |
|  |  |  | ☐ 6 = Brochure | | | | | | |
|  |  |  | ☐ 7 = School | | | | | | |
|  |  |  | ☐ 8 = Nurse/Doctor | | | | | | |
|  |  |  | ☐ 9 = Internet/social media | | | | | | |
|  |  |  | ☐ 10 = Parents/Family | | | | | | |
|  |  |  | ☐ 11 = Others, please specify ________________ | | | | | | |
| 3 | | Have you ever had worms (Roundworms, Whipworms, and Hookworms) yourself? | ☐ 0 = No | | | | | | |
|  |  |  | ☐ 1 = Yes | | | | | | |
|  |  |  | ☐ 2 = Don’t know | | | | | | |
| 4 | | Has somebody you know already had worms (Roundworms, Whipworms, and Hookworms)? | ☐ 0 = No | | | | | | |
|  |  |  | ☐ 1 = Yes | | | | | | |
|  |  |  | ☐ 2 = Don’t know | | | | | | |
| **D. TRANSMISSION, SYMPTOMS AND TREATMENT OF INTESTINAL WORMS** | | | | | | | | | |
| 1 | | How can you get infected with worms (Roundworms, Whipworms, and Hookworms)? Name all the possibilities you might know | ☐ 1 = Swimming in the canal/river  ☐ 2 = Walk Barefoot  ☐ 3 = Eat dirty food  ☐ 4 = Fishing  ☐ 5 = Long and dirty fingernails  ☐ 6 = Flies landing on wounds  ☐ 7 = Playing in dirty places  ☐ 8 = Mosquito bite  ☐ 9 = Dirty hands  ☐ 10 = Playing with soil | | | | | | |
|  |  |  | ☐ 11 = Others, please specify_________________________  ☐ 12 = Don’t know | | | | | | |
| 2 | | Do you think intestinal worms can cause serious disease? | ☐ 1= Yes | | | | | | |
|  |  |  | ☐ 2= No | | | | | | |
|  |  |  | ☐ 3= Don’t know | | | | | | |
| 3 | | What happens if people are infected with intestinal worms? (You can choose more than one answer) | ☐ 1= Feeling tired | | | | | | |
|  |  |  | ☐ 2= Blindness | | | | | | |
|  |  |  | ☐ 3= High blood pressure | | | | | | |
|  |  |  | ☐ 4= Diarrhea | | | | | | |
|  |  |  | ☐ 5= Overweight | | | | | | |
|  |  |  | ☐ 6= Slow growth | | | | | | |
|  |  |  | ☐ 7= Can’t concentrate at school | | | | | | |
|  |  |  | ☐ 8= Fever | | | | | | |
|  |  |  | ☐ 9= Poor appetite | | | | | | |
|  |  |  | ☐ 10= Belly ache | | | | | | |
|  |  |  | ☐ 11= Others, please describe _____________ | | | | | | |
|  |  |  | ☐ 12= Don’t Know | | | | | | |
| 4 | | How can you prevent/ avoid a worm infection? (Choose all correct answers) | ☐ 1= Washing fruit & vegetables if eating them raw  ☐ 2= Sleeping under a mosquito net  ☐ 3= Regular cutting of nails  ☐ 4= Eating too much  ☐ 5= Cover food  ☐ 6= Using the latrine  ☐ 7= Washing hands after toilet  ☐ 8= Always wear shoes or sandals  ☐ 9= Washing hands before eating  ☐ 10= Doing enough exercise  ☐ 11= Others, please describe __________________  ☐ 12= Don’t Know | | | | | | |
| 5 | | Do you think intestinal worm infection can be treated? | ☐ 0= No (Go to D7) | | | | | | |
|  |  |  | ☐ 1= Yes | | | | | | |
|  |  |  | ☐ 2= Don’t know (Go to D7) | | | | | | |
| 6 | | If yes, where can you go for treatment?  (You can choose more than one answer) | ☐ 1= School | | | | | | |
|  |  |  | ☐ 2= Local Clinic | | | | | | |
|  |  |  | ☐ 3= Hospital | | | | | | |
|  |  |  | ☐ 4= My parents | | | | | | |
|  |  |  | ☐ 5= Village doctor | | | | | | |
|  |  |  | ☐ 6= Don’t know | | | | | | |
| 7 | | If you take medicine for intestinal worms, will you be cured forever? | ☐ 0 = No | | | | | | |
|  |  |  | ☐ 1 = Yes | | | | | | |
|  |  |  | ☐ 2 = Don’t know | | | | | | |
| **E. ATTITUDE RELATED TO INTESTINAL WORMS** | | | | | | | | | |
| 1 | | Do you think you are at risk of getting worms (Roundworms, Whipworms, and Hookworms)? | | ☐ 0= No (Go to E2) | | | | | |
|  |  |  |  | ☐ 1= Yes | | | | | |
|  |  |  |  | ☐ 2= Don’t know (Go to E2) | | | | | |
| 1.1 | | If yes, what is your risk of getting worms? | | ☐ 0= None  ☐ 1= Low  ☐ 2= Medium  ☐ 3= High | | | | | |
| 2 | | Will you be worried anxious if you got intestinal worms (Roundworms, Whipworms, and Hookworms)? | | ☐ 0 = No  ☐ 1 = Yes  ☐ 2 = Don’t know | | | | | |
| 3 | | What do you think is the chance that your neighbour will be infected with intestinal worms? | | 0= None  1= Low  2= Medium  3= High  ☐ 4= Don’t know | | | | | |
| 4 | | How bad do you think STH or intestinal worms are as a disease in your village? | | ☐ 0= Not severe  ☐ 1= Low severity  ☐ 2= Medium severity  ☐ 3= High severity  ☐ 4= Don’t know | | | | | |
| 5 | | STH is a problem in your village. | | 0= Strongly disagree  1=Disagree  2= Agree  3= Strongly Agree | | | | | |
| **F. HEALTH EDUCATION RELATED TO INTESTINAL WORM** | | | | | | | | | |
| 1 | | Has the teacher already told you about intestinal worms? | | ☐ 0= No  ☐ 1= Yes  ☐ 2= Don’t know | | | | | |
| 2 | | Have you watched a video (in social media or Youtube) at school on intestinal worms (Roundworms, Whipworms and Hookworms)? | | ☐ 0= No  ☐ 1= Yes  ☐ 2= Don’t know | | | | | |
| 3 | | Have you ever done an assignment on intestinal worms? | | ☐ 0= No  ☐ 1= Yes  ☐ 2= Don’t know | | | | | |
| 4 | | Have you told your parents, sisters and brothers about intestinal worms? | | ☐ 0= No  ☐ 1= Yes  ☐ 2= Don’t know | | | | | |
| **G. BEHAVIOUR RELATED TO INTESTINAL WORMS** | | | | | | | | | |
| **Instruction:** Please put check mark (√) on the answer. | | | | | **Always (1)**  (10/10) | **Often (2)**  (7-9 out of 10 times) | **Sometimes (3)**  (4-6 out of 10 times) | **Rarely (4)**  (1-3 out of 10 times) | **Never (5)**  (0/10) |
| 1 | Do you wash hands after toilet use? | | | |  |  |  |  |  |
| 2 | Do you wash hands before eating? | | | |  |  |  |  |  |
| 3 | Do you wash hands after eating? | | | |  |  |  |  |  |
| 4 | How often do use soap when washing hands? | | | |  |  |  |  |  |
| 5 | Do you wear shoes or sandals when you go outside? | | | |  |  |  |  |  |
| 6 | Do you wash or peel fruit before you eat? | | | |  |  |  |  |  |
| 7 | Do you eat raw or un-boiled vegetables? | | | |  |  |  |  |  |
| 8 | Where does your family usually defecate? Choose one answer. | | | | ☐ 1= Home latrine | | | | |
|  |  |  |  |  | ☐ 2= Public latrine | | | | |
|  |  |  |  |  | ☐ 3= Field | | | | |
|  |  |  |  |  | ☐ 4= River | | | | |
|  |  |  |  |  | ☐ 5= Other place, specify: ___________________ | | | | |
| 9 | Where do you usually defecate? Choose one answer. | | | | ☐ 1= Home latrine | | | | |
|  |  |  |  |  | ☐ 2= Public latrine | | | | |
|  |  |  |  |  | ☐ 3= Field | | | | |
|  |  |  |  |  | ☐ 4= River | | | | |
|  |  |  |  |  | ☐ 5= Other place, specify: ___________________ | | | | |
