# Supplementary material for: Examining the Acceptability of Helminth Education Packages “Magic Glasses Lower Mekong” and “Magic Glasses Opisthorchiasis” and Their Impact on Knowledge, Attitudes, and Practices Among Schoolchildren in the Lower Mekong Basin: Protocol for a Cluster Randomized Controlled Trial
Source: JMIR Res Protoc. 2024 Sep 16;13:e55290. doi: 10.2196/55290 (PMC11443236; doi:10.2196/55290)
Supplement: Multimedia Appendix 4 [file resprot_v13i1e55290_app4.docx]

**Multimedia Appendix. MGO acceptability questionnaire (adapted from Sekhon et al. 2022) for the “Magic Glasses Lower Mekong” and “Magic Glasses Opisthorchiasis” cluster-randomized controlled trial.**

| **A. IDENTIFICATION** Date of survey (yyyy/mm/dd): _________-___-___  To be filled in by research team | | | | |
| --- | --- | --- | --- | --- |
| **1** | Village (Name and Code) | | | _______________________\|__\|__\| |
| **2** | School (Name and Code) | | | _______________________\|__\|__\| |
| **3** | Grade | | |  |
| **4** | Class/Section | | | \|__\|__\| |
| **5** | Student Number | | | \|__\|__\| |
| **B. PERSONAL INFORMATION**  To be filled in by children: Please fill in this questionnaire truthfully with the help of your teacher. All responses will be kept confidential and will not be used against you in any way. The info will be used for research purposes only*.* | | | | |
| 1 | Last name/Family name | | |  |
| 2 | First name/Given name | | |  |
| 3 | Sex | | | ☐ 1 – Male ☐ 2 – Female |
| 4 | Date of Birth (yyyy-mm-dd)  If you can’t remember, use 6 for the month/15 for the day of the month | | | \|__\|__ \|__\|__\| / \|__\|__\| / \|__\|__\| |
| **C. Affective attitude (measuring how an individual feels about the intervention)** | | | | |
| 1.1 | | “I liked the cartoon” | ☐ 1= strongly disagree  ☐ 2= disagree  ☐ 3= no opinion  ☐ 4= agree  ☐ 5= strongly agree | |
| 1.2 | | What did you like or dislike about the cartoon? | ------------------------------------------------------------------------------------------------------------------------------------------------------------------------------------------------------------------------------------ | |
| 2.1 | | “I thought the cartoon was funny” | ☐ 1= strongly disagree  ☐ 2= disagree  ☐ 3= no opinion  ☐ 4= agree  ☐ 5= strongly agree | |
| 2.2 | | What did you find funny or not funny about the cartoon? | ---------------------------------------------------------------------------------------------------------------------------------------------------------------------------------------------------------------------------------------------------- | |
| 3.1 | | “I thought the cartoon was scary” | ☐ 1= strongly disagree  ☐ 2= disagree  ☐ 3= no opinion  ☐ 4= agree  ☐ 5= strongly agree | |
| 3.2 | | What did you find scary or not scary about the cartoon? | ---------------------------------------------------------------------------------------------------------------------------------------------------------------------------------------------------------------------------------------------------- | |
| 4.1 | | “I thought the cartoon was entertaining” | ☐ 1= strongly disagree  ☐ 2= disagree  ☐ 3= no opinion  ☐ 4= agree  ☐ 5= strongly agree | |
| 4.2 | | What did you find entertaining or not entertaining about the cartoon? | ---------------------------------------------------------------------------------------------------------------------------------------------------------------------------------------------------------------------------------------------------- | |
| 5.1 | | “I related to the cartoon” | ☐ 1= strongly disagree  ☐ 2= disagree  ☐ 3= no opinion  ☐ 4= agree  ☐ 5= strongly agree | |
| 5.2 | | What parts of the cartoon did you relate to? | ---------------------------------------------------------------------------------------------------------------------------------------------------------------------------------------------------------------------------------------------------- | |
| 6.1 | | The characters and the setting of the cartoon made me think of my village” | ☐ 1= strongly disagree  ☐ 2= disagree  ☐ 3= no opinion  ☐ 4= agree  ☐ 5= strongly agree | |
| 6.2 | | What parts of the cartoon made you think of your village? | ---------------------------------------------------------------------------------------------------------------------------------------------------------------------------------------------------------------------------------------------------- | |
| 7.1 | | What was your favourite scene in the cartoon, and why? | ------------------------------------------------------------------------------------------------------------------------------------------------------------------------------------------------ | |
| **D. Burden (the amount of effort required to participate in the intervention)** | | | | |
| 1.1 | | “It was easy for me to watch the cartoon” | ☐ 1= strongly disagree  ☐ 2= disagree  ☐ 3= no opinion  ☐ 4= agree  ☐ 5= strongly agree | |
| 2.1 | | “It was easy for me to pay attention to the cartoon” | ☐ 1= strongly disagree  ☐ 2= disagree  ☐ 3= no opinion  ☐ 4= agree  ☐ 5= strongly agree | |
| 3.1 | | “I was bored watching the cartoon” | ☐ 1= strongly disagree  ☐ 2= disagree  ☐ 3= no opinion  ☐ 4= agree  ☐ 5= strongly agree | |
| 4.1 | | “I did not like having to watch the cartoon” | ☐ 1= strongly disagree  ☐ 2= disagree  ☐ 3= no opinion  ☐ 4= agree  ☐ 5= strongly agree | |
| **E. Ethicality (the extent to which the intervention has good fit with an individual’s value system)** | | | | |
| **E. ក្រមសីលធម៌ (វិសាលភាពនៃការអន្តរាគមន៏ដែលសមស្របទៅនឹងប្រព័ន្ធតម្លៃរបស់បុគ្គលម្នាក់ៗ)** | | | | |
| 1.1 | | “This cartoon would be appropriate for others my age to watch” | ☐ 1= strongly disagree  ☐ 2= disagree  ☐ 3= no opinion  ☐ 4= agree  ☐ 5= strongly agree | |
| 1.2 | | Why? | ---------------------------------------------------------------------------------------------------------------------------------------------------------------------------------------------------------------------------------------------------- | |
| 2.1 | | “My parents would let me watch this cartoon” | ☐ 1= strongly disagree  ☐ 2= disagree  ☐ 3= no opinion  ☐ 4= agree  ☐ 5= strongly agree | |
| 2.2 | | Why? | --------------------------------------------------------------------------------------------------------------------------------------------------------------------------- | |
| 3.1 | | “Other parents would let their children watch this cartoon” | ☐ 1= strongly disagree  ☐ 2= disagree  ☐ 3= no opinion  ☐ 4= agree  ☐ 5= strongly agree | |
| 3.2 | | Why? | ---------------------------------------------------------------------------------------------------------------------------------------------------------------------------------------------------------------------------------------------------- | |
| **F. Perceived effectiveness (the extent to which the intervention has achieved its purpose)** | | | | |
| 1.1 | | “The cartoon improved my knowledge of liver flukes” | ☐ 1= strongly disagree  ☐ 2= disagree  ☐ 3= no opinion  ☐ 4= agree  ☐ 5= strongly agree | |
| 2.1 | | “The cartoon taught me the risk of liver flukes” | ☐ 1= strongly disagree  ☐ 2= disagree  ☐ 3= no opinion  ☐ 4= agree  ☐ 5= strongly agree | |
| 3.1 | | “The cartoon made it clear how I can protect myself from liver flukes” | ☐ 1= strongly disagree  ☐ 2= disagree  ☐ 3= no opinion  ☐ 4= agree  ☐ 5= strongly agree | |
| 4.1 | | “The cartoon made me want to avoid eating uncooked fish” | ☐ 1= strongly disagree  ☐ 2= disagree  ☐ 3= no opinion  ☐ 4= agree  ☐ 5= strongly agree | |
| **G. Self-efficacy (the participant’s confidence that they can perform behaviour(s) required to participate in the intervention** | | | | |
| 1.1 | | “I will be able to avoid eating uncooked fish” | ☐ 1= strongly disagree  ☐ 2= disagree  ☐ 3= no opinion  ☐ 4= agree  ☐ 5= strongly agree | |
| 1.2 | | Why? | --------------------------------------------------------------------------------------------------------------------------------------------------------------------------- | |
| 2.1 | | “I/my family will be able to cook the fish” | ☐ 1= strongly disagree  ☐ 2= disagree  ☐ 3= no opinion  ☐ 4= agree  ☐ 5= strongly agree | |
| 2.2 | | Why? | --------------------------------------------------------------------------------------------------------------------------------------------------------------------------- | |
| 3.1 | | “Cooking fish will be difficult for me/my family” | ☐ 1= strongly disagree  ☐ 2= disagree  ☐ 3= no opinion  ☐ 4= agree  ☐ 5= strongly agree | |
| 3.2 | | Why? | --------------------------------------------------------------------------------------------------------------------------------------------------------------------------- | |
| 1.1 | | “I thought that the cartoon was acceptable” | ☐ 1= strongly disagree  ☐ 2= disagree  ☐ 3= no opinion  ☐ 4= agree  ☐ 5= strongly agree | |
| 2.1 | | “I would watch the cartoon again” | ☐ 1= strongly disagree  ☐ 2= disagree  ☐ 3= no opinion  ☐ 4= agree  ☐ 5= strongly agree | |
| 3.1 | | “I think others would watch the cartoon again” | ☐ 1= strongly disagree  ☐ 2= disagree  ☐ 3= no opinion  ☐ 4= agree  ☐ 5= strongly agree | |
| 4.1 | | “I would tell others to watch the cartoon” | ☐ 1= strongly disagree  ☐ 2= disagree  ☐ 3= no opinion  ☐ 4= agree  ☐ 5= strongly agree | |
