# Supplementary material for: Examining the Acceptability of Helminth Education Packages “Magic Glasses Lower Mekong” and “Magic Glasses Opisthorchiasis” and Their Impact on Knowledge, Attitudes, and Practices Among Schoolchildren in the Lower Mekong Basin: Protocol for a Cluster Randomized Controlled Trial
Source: JMIR Res Protoc. 2024 Sep 16;13:e55290. doi: 10.2196/55290 (PMC11443236; doi:10.2196/55290)
Supplement: Multimedia Appendix 5 [file resprot_v13i1e55290_app5.docx]

**Multimedia Appendix. MGLM acceptability questionnaire (adapted from Sekhon et al. 2022) for the “Magic Glasses Lower Mekong” and “Magic Glasses Opisthorchiasis” cluster-randomized controlled trial.**

| **A. IDENTIFICATION** Date of survey (yyyy/mm/dd): ________-___-___  To be filled in by research team: | | |
| --- | --- | --- |
| **1** | Village (Name and Code) | _______________________\|__\|__\| |
| **2** | School (Name and ID) | _______________________\|__\|__\| |
| **3** | Grade |  |
| **4** | Class/Section |  |
| **5** | Individual ID | \|__\|__\| |
| **B. PERSONAL INFORMATION** | | |
| 1 | Last name/Family name |  |
| 2 | First name/Given name |  |
| 3 | Sex | ☐ 1 – Male ☐ 2 – Female |
| 4 | Date of Birth (yyyy-mm-dd)  If you can’t remember, use 6 for the month/15 for the day of the month | \|__\|__ \|__\|__\| / \|__\|__\| / \|__\|__\| |
| **C. Affective attitude (measuring how an individual feels about the intervention)** | | |
| 1.1 | “I liked the cartoon” | ☐ 1= strongly disagree  ☐ 2= disagree  ☐ 3= no opinion  ☐ 4= agree  ☐ 5= strongly agree |
| 1.2 | What did you like or dislike about the cartoon? | ------------------------------------------------------------------------------------------------------------------------------------------------------------------------------------------------------------------------------------ |
| 2.1 | “I thought the cartoon was funny” | ☐ 1= strongly disagree  ☐ 2= disagree  ☐ 3= no opinion  ☐ 4= agree  ☐ 5= strongly agree |
| 2.2 | What did you find funny or not funny about the cartoon? | ------------------------------------------------------------------------------------------------------------------------------------------------------------------------------------------------------------------------------------ |
| 3.1 | “I thought the cartoon was scary” | ☐ 1= strongly disagree  ☐ 2= disagree  ☐ 3= no opinion  ☐ 4= agree  ☐ 5= strongly agree |
| 3.2 | What did you find scary or not scary about the cartoon? | ------------------------------------------------------------------------------------------------------------------------------------------------------------------------------------------------------------------------------------ |
| 4.1 | “I thought the cartoon was entertaining” | ☐ 1= strongly disagree  ☐ 2= disagree  ☐ 3= no opinion  ☐ 4= agree  ☐ 5= strongly agree |
| 4.2 | What did you find entertaining or not entertaining about the cartoon? | ------------------------------------------------------------------------------------------------------------------------------------------------------------------------------------------------------------------------------------ |
| 5.1 | “I related to the cartoon” | ☐ 1= strongly disagree  ☐ 2= disagree  ☐ 3= no opinion  ☐ 4= agree  ☐ 5= strongly agree |
| 5.2 | What parts of the cartoon did you relate to? | ------------------------------------------------------------------------------------------------------------------------------------------------------------------------------------------------------------------------------------ |
| 6.1 | The characters and the setting of the cartoon made me think of my village” | ☐ 1= strongly disagree  ☐ 2= disagree  ☐ 3= no opinion  ☐ 4= agree  ☐ 5= strongly agree |
| 6.2 | What parts of the cartoon made you think of your village? | ------------------------------------------------------------------------------------------------------------------------------------------------------------------------------------------------------------------------------------ |
| 7.1 | What was your favourite scene in the cartoon, and why? | ------------------------------------------------------------------------------------------------------------------------------------------------------------------------------------------------------------------------------------ |
| **D. Burden (the amount of effort required to participate in the intervention)** | | |
| 1.1 | “It was easy for me to watch the cartoon” | ☐ 1= strongly disagree  ☐ 2= disagree  ☐ 3= no opinion  ☐ 4= agree  ☐ 5= strongly agree |
| 2.1 | “It was easy for me to pay attention to the cartoon” | ☐ 1= strongly disagree  ☐ 2= disagree  ☐ 3= no opinion  ☐ 4= agree  ☐ 5= strongly agree |
| 3.1 | “I was bored watching the cartoon” | ☐ 1= strongly disagree  ☐ 2= disagree  ☐ 3= no opinion  ☐ 4= agree  ☐ 5= strongly agree |
| 4.1 | “I did not like having to watch the cartoon” | ☐ 1= strongly disagree  ☐ 2= disagree  ☐ 3= no opinion  ☐ 4= agree  ☐ 5= strongly agree |
| **E. Ethicality (the extent to which the intervention has good fit with an individual’s value system)** | | |
| 1.1 | “This cartoon would be appropriate for others my age to watch” | ☐ 1= strongly disagree  ☐ 2= disagree  ☐ 3= no opinion  ☐ 4= agree  ☐ 5= strongly agree |
| 1.2 | Why? | ------------------------------------------------------------------------------------------------------------------------------------------------------------------------------------------------------------------------------------ |
| 2.1 | “My parents would let me watch this cartoon” | ☐ 1= strongly disagree  ☐ 2= disagree  ☐ 3= no opinion  ☐ 4= agree  ☐ 5= strongly agree |
| 2.2 | Why? | --------------------------------------------------------------------------------------------------------------------------------------------------------- |
| 3.1 | “Other parents would let their children watch this cartoon” | ☐ 1= strongly disagree  ☐ 2= disagree  ☐ 3= no opinion  ☐ 4= agree  ☐ 5= strongly agree |
| 3.2 | Why? | ------------------------------------------------------------------------------------------------------------------------------------------------------------------------------------------------------------------------------------ |
| **F. Perceived effectiveness (the extent to which the intervention has achieved its purpose)** | | |
| 1.1 | “The cartoon improved my knowledge of worms” | ☐ 1= strongly disagree  ☐ 2= disagree  ☐ 3= no opinion  ☐ 4= agree  ☐ 5= strongly agree |
| 2.1 | “The cartoon taught me the risk of worms” | ☐ 1= strongly disagree  ☐ 2= disagree  ☐ 3= no opinion  ☐ 4= agree  ☐ 5= strongly agree |
| 3.1 | “The cartoon made it clear how I can protect myself from worms when outside” | ☐ 1= strongly disagree  ☐ 2= disagree  ☐ 3= no opinion  ☐ 4= agree  ☐ 5= strongly agree |
| 4.1 | “The cartoon made it clear how I can protect myself from worms when making or eating food” | ☐ 1= strongly disagree  ☐ 2= disagree  ☐ 3= no opinion  ☐ 4= agree  ☐ 5= strongly agree |
| **G. Self-efficacy (the participant’s confidence that they can perform behaviour(s) required to participate in the intervention** | | |
| 1.1 | “I will be able to wash my hands before eating” | ☐ 1= strongly disagree  ☐ 2= disagree  ☐ 3= no opinion  ☐ 4= agree  ☐ 5= strongly agree |
| 1.2 | Why? | ------------------------------------------------------------------------------------------------------------------ |
| 2.1 | “I will be able to wash or cook fruit and vegetables before I eat them” | ☐ 1= strongly disagree  ☐ 2= disagree  ☐ 3= no opinion  ☐ 4= agree  ☐ 5= strongly agree |
| 2.2 | Why? | ------------------------------------------------------------------------------------------------------------------ |
| 3.1 | “I will be able to wear shoes/slippers when outside” | ☐ 1= strongly disagree  ☐ 2= disagree  ☐ 3= no opinion  ☐ 4= agree  ☐ 5= strongly agree |
| 3.2 | Why? | ------------------------------------------------------------------------------------------------------------------ |
| **H. General acceptability** | | |
| 1.1 | “I thought that the cartoon was acceptable” | ☐ 1= strongly disagree  ☐ 2= disagree  ☐ 3= no opinion  ☐ 4= agree  ☐ 5= strongly agree |
| 2.1 | “I would watch the cartoon again” | ☐ 1= strongly disagree  ☐ 2= disagree  ☐ 3= no opinion  ☐ 4= agree  ☐ 5= strongly agree |
| 3.1 | “I think others would watch the cartoon again” | ☐ 1= strongly disagree  ☐ 2= disagree  ☐ 3= no opinion  ☐ 4= agree  ☐ 5= strongly agree |
| 4.1 | “I would tell others to watch the cartoon” | ☐ 1= strongly disagree  ☐ 2= disagree  ☐ 3= no opinion  ☐ 4= agree  ☐ 5= strongly agree |
